# Molecular Survey and Identification of *Campylobacter* spp. in Layer Farms in Central Ethiopia

**DOI:** 10.3390/tropicalmed7020031

**Published:** 2022-02-18

**Authors:** Behailu Assefa Wayou, Gezahegne Mamo Kassa, Teshale Sori, Alessandra Mondin, Claudia Maria Tucciarone, Mattia Cecchinato, Daniela Pasotto

**Affiliations:** 1 Department of Veterinary Microbiology Immunology and Public Health, College of Veterinary Medicine and Agriculture, Addis Ababa University, Bishoftu P.O. Box 34, Ethiopia; newbaye@gmail.com (B.A.W.); gezahegnemamo@gmail.com (G.M.K.); 2 Department of Clinical Studies, College of Veterinary Medicine and Agriculture, Addis Ababa University, Bishoftu P.O. Box 34, Ethiopia; teshalesori2002@yahoo.com; 3 Department of Animal Medicine, Production and Health, University of Padua, 35020 Legnaro, Italy; alessandra.mondin@unipd.it (A.M.); mattia.cecchinato@unipd.it (M.C.); daniela.pasotto@unipd.it (D.P.); 4 Department of Comparative Biomedicine and Food Science, University of Padua, 35020 Legnaro, Italy

**Keywords:** campylobacteriosis, public health, poultry, layers

## Abstract

Few data are available on *Campylobacter* spp. presence in chickens in Ethiopia. Due to its importance for both the poultry sector and public health, a sampling activity was planned to evaluate *Campylobacter* spp. presence in layer farms in Bishoftu and Mojo, Central Ethiopia. Twenty cloacal pooled samples were collected and tested with molecular assays for detection and Sanger-sequenced for species identification. As a secondary aim, samples were also tested for *Salmonella* spp. by PCR, and all samples were negative. On the other hand, 70% of cloacal swab pools were positive for *Campylobacter* spp.: 71.4% of the positive samples belonged to *C. jejuni* species, 21.4% to *C. avium* and 7.1% to *C. helveticus*. *Campylobacter* spp. was identified in almost all farms regardless of farm and flock size, age and hybrid types of the birds and antimicrobial treatment. *Campylobacter jejuni* is a common finding in chickens, whereas species such as *C. avium* and *C. helveticus* were newly reported in Ethiopia, revealing a variability that needs to be monitored in light of the public health significance of this pathogen.

## 1. Introduction

Poultry meat and eggs provide nutritionally beneficial food containing high quality protein, although they may present some risks in terms of food safety, especially in regions where the productive sector is rapidly expanding [1]. In Ethiopia, the vast majority of domestic fowl are reared in villages, under a traditional scavenging system that allows poultry to be owned by almost 60% of households, and only a small percentage are reared intensively [2], although this type of production is consistently increasing. However, the lack of an organized poultry health service and limited access to veterinary assistance, including both diagnosis and vaccination, are inevitably linked to high mortality and disease occurrence rates in birds [1]. Poultry pathogens not only impact productivity and animal health but they are intimately connected to food safety and human health, especially when chickens and people share the same environment. Exposure to animals was indeed proven as one of the major risk factors for diarrhea in children under 5 years of age in Ethiopia [3].

As a matter of fact, among the most common foodborne zoonotic agents, *Campylobacter* spp. is a bacterium that commonly inhabits the gastrointestinal tract of many domestic animals, especially chickens [4]. The *Campylobacter* genus currently comprises 32 species and 9 sub-species [5], with *C. jejuni* being the most common isolate in poultry, followed by *C. coli* and *C. lari* [6].

There are several pathways for *Campylobacter* spp. colonization of poultry flocks, including the persistence from previous productive cycles, horizontal transfer from other animals (wild or domestic), insects, fomites and contaminated feed and water. Once a flock is colonized with *Campylobacter* spp., feed, water and litter quickly become contaminated, and the infection reaches high prevalence among the birds. Even though it is highly prevalent in commercial, organic and free-range poultry farms, birds that access an environment where no disinfection can be applied and that are reared for longer periods before slaughtering are at higher risk [7]; as in backyard flocks. There, they can be exposed to wild birds, which are also reservoirs of *Campylobacter* spp., although with a lower prevalence likely linked to the sparse animal density [8]. Flock size, house number, the use of untreated water and disposal of poultry wastes on the farm are other risk factors for *Campylobacter* spp. colonization [9]. Although poultry can act just as a carrier without clinical symptoms, they serve as a potential source for human infection [10]. In fact, campylobacteriosis is the most frequently reported gastroenteric infection in humans in Europe, and the most common source appears to be chicken and turkey meat [11]. Among bacterial zoonotic agents, *Campylobacter* spp. is the most important food safety hazard, and together with *Salmonella* spp., they account for more than 90 percent of all reported cases of bacteria-related food poisonings [11].

*Salmonella* spp. is, in fact, the second most important zoonotic cause of gastrointestinal disease in humans [11] due to contaminated food, mainly poorly cooked meat, eggs, water or contact with droppings or animals, that can be asymptomatic reservoirs [12]. Mammals, reptiles, amphibians, fish, shellfish and birds are hosts of *Salmonella* spp. [13], but chickens and turkeys are the most frequently acknowledged sources of infection. In fact, many factors favor enteric colonization in poultry, such as the bacterium resistance to the gastric passage, the age of the birds, the use of antimicrobial drugs, farming conditions and stressors, health status and immunosuppression. Additionally, for *Salmonella* spp., when introduced into a flock, a high percentage of animals are rapidly colonized [14].

In Ethiopia, *Salmonella* spp. was recently detected in different animal products [15,16,17], with a parallel increase in antimicrobial resistance against most commonly used drugs [18,19,20,21], both in animals and humans.

Campylobacteriosis and salmonellosis are leading foodborne zoonoses in Ethiopia, and they are often the cause of gastrointestinal infections in humans, sustaining serious diseases in children [3,22]. Immunosuppression, malnutrition and old age are other risk factors for human infection [23]. In most cases, campylobacteriosis and salmonellosis are self-limiting infections; however, in immunocompromised or critically ill patients, antimicrobial therapy may be required [24].

It was reported that chickens and their meat have a higher *Campylobacter* spp. prevalence compared to other farm animal and meat products [25], and birds can also act as a source of infection by direct and domestic exposure [3]. Poultry workers in farms, slaughterhouses and meat processing facilities are also at risk of *Campylobacter* spp. and *Salmonella* spp. infections [26] through direct contact with infected birds, their products and contaminated materials or environments [10].

The present study aimed primarily to detect and identify *Campylobacter* species in layer farms in Bishoftu and Mojo, located in the Oromia region in Central Ethiopia, and samples were also tested for *Salmonella* species.

## 2. Materials and Methods

The study was centered around Bishoftu and Mojo, which are located in the Oromia region, Central Ethiopia, in the proximity of Addis Ababa. These two cities were expressly selected based on the large number of both intensive and backyard layer farms located in the surrounding area.

Cloacal swabs from 10 hens were collected from each visited layer farm and air-dried to avoid mold growth before being pooled. Multiple sheds were sampled when present in the farms. Data about the farms were recorded in a comprehensive database of the number of sheds and birds, age and genetic type, the overall mortality rate (total number of recovered dead birds in the ongoing cycle), administered treatments, vaccination protocol, clinical signs and lesions, when present. Samples were briefly refrigerated; then they were packed and shipped to the Animal Medicine, Production and Health (MAPS) Department at the University of Padua (Legnaro, Italy) for molecular analyses. Swabs were resuspended in 2 mL of sterile PBS and carefully vortexed before using a 200 µL aliquot for DNA extraction with the QIAamp DNA Mini Kit (Qiagen, Hilden, Germany). Extracted samples were stored at −20 °C until further analyses.

Samples were tested with molecular assays to determine the occurrence of *Campylobacter* spp. and *Salmonella* spp. For *Campylobacter* spp. detection, a genus-specific PCR was performed as described by Linton et al. (1996) [27] targeting the 16S rRNA gene. For *Salmonella* spp. detection, a genus-specific PCR was performed, as described by Chiu et al. (1996) [28], targeting the chromosomal *invA* and plasmid *spvC* genes for rapid genus identification and evaluation of virulence, respectively. Both reactions were performed using the Thermo Scientific™ (Waltham, MA, USA) Phire Hot Start II DNA Polymerase kit (Invitrogen™, Waltham, MA, USA) on an Applied Biosystems 2720 Thermal Cycler (Applied Biosystems, Waltham, MA, USA), including sterile water as a negative control and strains of *Salmonella* spp. (ATCC 700623™, Manassas, VA, USA) and *Campylobacter jejuni* (ATCC 33560™) as positive controls. Amplicon presence and specificity were examined by agar gel electrophoresis in SYBR™ Safe-stained (Invitrogen™, Waltham, MA, USA) agar gel.

For species identification, positive samples were Sanger-sequenced with the same primer pairs used for amplification in both directions [27,28]. PCR products were shipped to the external sequencing service of Macrogen Spain (Madrid, Spain) for purification and Sanger sequencing. Chromatogram quality was inspected with FinchTV software (Geospiza Inc., Seattle, WA, USA) and forward and reverse sequences were assembled in consensus sequences using ChromasPro 2.1.8 software (Technelysium Pty Ltd., Helensvale, QLD, Australia). Nucleotide sequences were evaluated by BLAST search for preliminary species identification [29]. Sequences were deposited in Genbank and aligned to a reference dataset in MEGA X [30] software for phylogenetic analyses. A phylogenetic tree was reconstructed using the Maximum Likelihood method, and branch support was calculated by performing 1000 bootstrap replicates.

A Fisher’s exact probability test was performed to evaluate the association between positivity and antimicrobial treatment or clinical signs, whereas a Student’s *t*-test was used to evaluate the association between age or flock size and positivity (http://vassarstats.net; last accessed on 17 December 2021) [31].

## 3. Results

A total of 20 pooled samples were collected from 15 layer farms located in Bishoftu (nine farms) and Mojo (six farms). In four farms, multiple sheds were sampled (Table 1). All farms reared birds indoors, mainly with a litter system, except for two farms using cage systems (one in Bishoftu, one in Mojo). Clinical sign presence was reported from four farms in Mojo and six farms in Bishoftu (10 farms in total) (Table 1). The administered treatments ranged from none or vitamin supplementation (eight farms) to antimicrobial drugs (oxytetracycline, sulfadiazine, norfloxacin) (six farms), diclazuril and amprolium hydrochloride coccidiostats (one farm). The hybrid type of the birds was Bovans Brown laying hens in nine farms and Lohmanns laying hens in six farms; the bird’s age ranged from 3 months to 1 year (mean 9.9 months). The population size of the farms ranged from 150 to 12,000 birds (mean 3317) (Table 1). Vaccination was performed in all farms: the vaccination protocol varied among the farms, but it generally included vaccines against Newcastle disease, infectious bronchitis, infectious bursal disease, Marek’s disease, fowl pox and fowl typhoid.

**Figure 1:**
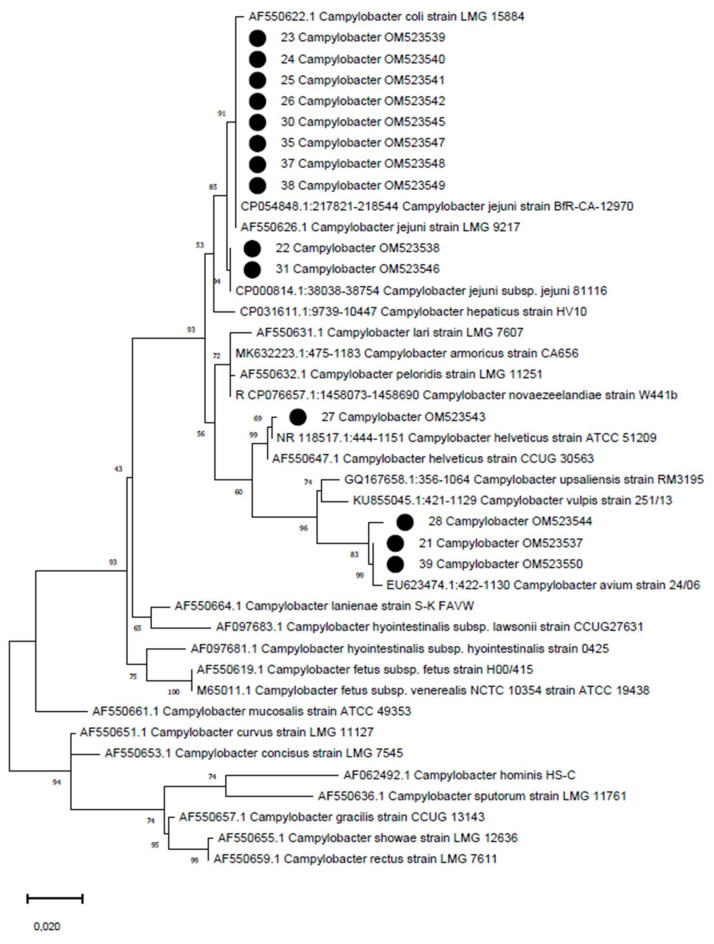
Phylogenetic tree reconstructed on 16S ribosomal RNA gene partial sequences of *Campylobacter*. The Ethiopian strains are marked with a black circle. The phylogenetic tree was reconstructed using the maximum likelihood method, with the Kimura 2-parameter and general time reversible model with discrete gamma distribution. Branch support is shown next to the branches.

All samples were negative for *Salmonella* spp., whereas 14 out of 20 samples (70.00%) were positive for *Campylobacter* spp. indicating the positivity of 12 out of 15 farms (80.00%). Species identification, initially performed by BLAST, revealed that 10 samples (71.43%) were positive for *C. jejuni* with 99.58–100% identity with reference strain BfR-CA-12970 (Acc. Num. CP054848.1). Three samples (21.43%) were positive for *C. avium* with 99.03–99.72% identity with reference strain 24/06 (Acc. Num. EU623474.1), and 1 sample (7.14%) was positive for *C. helveticus* with 99.86% of identity with reference strain ATCC 51209 (Acc. Num. NR_118517.1). Sequences were deposited under accession numbers OM523537-OM523550 and species identification was confirmed by phylogenetic analysis (Figure 1) reconstructed with the reference sequence dataset proposed in Supplementary Table S1.

Half of the negative samples (3/6) were collected from farms where more than one shed was sampled (farms n. 3, 6, 13, 15; Table 1). In particular, *C. helveticus* was detected in one shed of farm 15, where multiple sheds were sampled (three sheds), and the two other sheds were positive for *C. jejuni*. In two farms (farms 6 and 13), a shed was positive for *C. jejuni*, and the other was negative, and in another farm (farm 3), *Campylobacter* (*C. avium*) was detected only in one of the two sheds.

Two out of eight farms that did not declare the use of antimicrobial drugs were negative, whereas only one out of seven farms reporting the use of antimicrobial drugs or coccidiostats was negative (Table 1). However, there was no statistically significant association between drug administration and positivity for *Campylobacter* spp. (*p* = 0.55).

One out of five farms not reporting clinical signs was negative for *Campylobacter* spp., whereas two out of ten farms with clinical signs were negative (Table 1), but there was no statistically significant association between clinical signs and positivity (*p* = 0.75). Seven out of eight farms reporting enteric signs were positive. The comparison between positive and negative farms in terms of age mean and flock size mean yielded a not statistically significant difference (*p* = 0.18; *p* = 0.41) (Table 1). The mean and median overall mortality rates in the farms enrolled in the study were 3.48% and 0.39% (range 0.00–19.23%), respectively, with higher rates in smaller farms (farms n. 1, 2, 3; <300 birds). The absence of mortality reported in some farms should be interpreted in light of the lack of rigorous recording methods and should be rather intended as an average rate of mortality based on the hybrid type of the birds, production and management in the farms in the absence of acute causes.

## 4. Discussion

In the current study, *Campylobacter* species were identified from most of the study farms (80.00%) regardless of the flock size, age of the birds, use of antimicrobial drugs and clinical sign presence. *Campylobacter* spp. is a commensal bacterium in poultry, and the findings of the present paper are in line with the previously reported data in Ethiopia [10,32,33,34], where it was commonly isolated from both egg-type and meat-type chickens [25]. Additionally, *C. jejuni* was the most frequently detected species, whereas the identification of the two other species is more interesting: in fact, there is no previous report of *C. avium* in poultry in Ethiopia, whereas *C. helveticus* was considered a risk related particularly to cats and dogs, which generally act as reservoirs of subclinical infection [35]. However, the scarce data about the circulation of these species should be imputed to the lack of diagnostic surveys rather than to their absence in the field, highlighting the usefulness of the present study.

All farms were negative for *Salmonella* spp. despite the previous detections in poultry farms in central Ethiopia [17]. A decrease in the susceptibility of *Salmonella* spp. was indicated for some of the antimicrobial drugs [36] (oxytetracycline, sulfadiazine, norfloxacin) that were reportedly administered in the farms enrolled in the study. This finding is not representative of the actual epidemiological situation of *Salmonella* spp., since the diagnostic method and the sampling type (dry swabs instead of enriched transport medium) used in this study are not the gold standard. The tests were performed on the same samples which were positive for *Campylobacter* spp., thus ruling out problems during the extraction phase or related to the conservation of the nucleic acids. However, it cannot be excluded that the bacterial titer in the samples could have been under the limit of detection of the biomolecular assay or that the intermittent shedding of *Salmonella* spp. could have led to negative results due to the single sampling in the farms. Environmental sampling or a larger bird sample size should be preferred when investigating *Salmonella* spp. Still, different isolation rates of *Salmonella* spp. are reported in Ethiopia, ranging from 4% to 16% [20,37], suggesting a certain variability in the prevalence.

Differently from *Salmonella* spp., for which a prevalence of around 7% in egg content was recorded in an Ethiopian study [38], egg consumption is not one of the main routes of human infection with *Campylobacter* spp. [39]. Nevertheless, its high prevalence in the farm could definitely lead to shell contamination, thus increasing the risk of ingestion, surface contamination and passage through the shell during handling or storage.

Unfortunately, the biomolecular nature of the survey did not allow us to further explore the patterns of antimicrobial susceptibility of the detected bacteria, especially in the farms where the use of antimicrobials was recorded. However, the wide antimicrobial use and the increasing spread of *Salmonella* spp. and *Campylobacter* spp. antimicrobial-resistant strains [40] highlight the importance of a continuous epidemiological update on these micro-organisms, resorting to different diagnostic approaches.

It was reported that, in Ethiopia, antimicrobial drugs can sometimes be administered in the absence of a clear diagnosis or susceptibility testing in response to various clinical signs or alterations [37]. Even if unrelated to the investigated pathogens, clinical signs have also been recorded in this study, attesting conditions where management could have been suboptimal. In fact, some of the farms involved in the present study reported clinical problems and the use of oxytetracycline, sulfadiazine and norfloxacin without disclosing the therapeutic indications and previous diagnostic tests.

In this study, when more than one shed in the farms was sampled, different scenarios were displayed, even in light of the same farming conditions: in two farms, one shed was positive for *C. jejuni*, and the other one was negative, whereas two other farms revealed the presence of different species of *Campylobacter* in different flocks. This finding is not unexpected since the coexistence of multiple strains was reported [41] and, in case of inadequate biosecurity measures, poor disinfection between cycles or contamination of the birds at the source, different bacteria could persist or be introduced in the same environment. Moreover, common biomolecular and Sanger-sequencing methods yield only single information about the detected species, generally revealing the most abundant species but potentially masking co-infections sustained by genetically similar pathogens. However, the quality of the sequences obtained in the present study supports the herein identified species. Despite this limitation, the presence of different species in the same farm was detected, revealing the heterogeneity of the circulating *Campylobacter* species.

Since the prevalence of *Campylobacter* spp. in poultry is determined by a wide range of factors, such as the contamination of the birds at the hatchery, life length, egg or meat production, farm and flock size, seasonality, litter, water, feed and management of environmental conditions, biosecurity might not be enough to avert flock colonization [42]. However, good hygiene and manufacturing practices should not be restricted to the farming level, and campylobacteriosis risk can be contained along the food production chain by limiting cross-contamination at slaughter and implementing strict disinfection procedures thus lowering the bacterial load. Regular monitoring on farms could also help to identify contaminated flocks that are destined for products restricted to cooking by thorough heat treatment for microbial inactivation [43,44]. The strict implementation of a cold chain during meat processing and distribution is another pivotal aspect for reducing microbial load on meat and preventing cross-contamination of other food products, tools and surfaces [43,44]. Still, this does not prevent contamination related to handling procedures at the farm level, where workers might be exposed to and carry *Campylobacter* spp. and *Salmonella* spp. if not well trained towards safety and good hygiene practices.

## 5. Conclusions

Herein lies the importance of even small-sized surveys in developing countries, such as Ethiopia, where intense diagnostic monitoring should be performed to support the growing poultry sector. The present study evidenced the presence and heterogeneity of different species of *Campylobacter*, drawing attention to the implementation of biosecurity measures, good hygiene practices and animal management. Conversely, the lack of *Salmonella* spp. detection suggests the importance of dedicated studies, employing classical microbiological approaches that would also allow the evaluation of the antimicrobial resistance profile of the detected pathogens.

A broader approach should result in regular updates of the epidemiological scenario, followed by the information and education of farmers and workers with regard to the conditions and requirements of their working and producing environment. A wider knowledge could also help the authorities to promote awareness in the consumers regarding good hygiene practices and food safety.

## Figures and Tables

**Table 1:** Information summary of data related to the sampled farms.

Farm	Shed	Location	N° of Birds	Age (Months)	Hybrid Type	Clinical Signs	Mortality (%) *	Treatment	*Campylobacter* spp.
1	-	Mojo 02	150	12	Bovans brown	Twisting of neck, diarrhea	13.33	Antimicrobial drug	*C. jejuni*
2	-	Mojo 01	200	12	Lohmann	-	0	Not administered	*C. jejuni*
3	1	Mojo 02	260	12	Bovans brown	Diarrhea, depression	19.23	Antimicrobial drugVitamin supply	*C. avium*
	2								-
4	-	Mojo 01	350	12	Lohmann	Depression, weakness	0	Not administered	*C. avium*
5	-	Mojo 02	450	12	Bovans brown	Diarrhea, swollen eyes	0.44	Antimicrobial drugVitamin supply	-
6	1	Mojo 01	500	12	Lohmann	-	0	Not administered	-
	2			7					*C. jejuni*
7	-	Bishoftu 05	570	10	Lohmann	Diarrhea, twisting of the neck	5.61	Antimicrobial drug	*C. jejuni*
8	-	Bishoftu 05	800	5	Bovans brown	Dyspnea	0.12	Vitamin supply	-
9	-	Bishoftu 09	1800	3	Lohmann	-	0.83	Vitamin supply	-
10	-	Bishoftu 01	2500	12	Lohmann	-	4	Antimicrobial drugVitamin supply	*C. jejuni*
11	-	Bishoftu 05	3000	5	Bovans brown	-	0.13	Vitamin supply	*C. jejuni*
12	-	Bishoftu 09	3000	3	Bovans brown	Diarrhea, depression	0.33	AnticoccidialVitamin supply	*C. jejuni*
13	1	Bishoftu 05	5000	12	Bovans brown	Diarrhea, twisting of the neck, leg paralysis	1.00	Vitamin supply	*C. jejuni*
	2								-
14	-	Bishoftu 01	6000	9	Bovans brown	Torticollis, swollen eyes, diarrhea, feather loss	3.33	Vitamin supply	*C.avium*
15	1	Bishoftu 05	12,000	12	Bovans brown	Swollen eyes, eye discharge, dyspnea, salivation	0.33	Antimicrobial drugVitamin supply	*C. jejuni*
	2					Swollen vent and eyes, eye discharge, dyspnea, salivation			*C. jejuni*
	3					Diarrhea, weakness/listlessness, depression			*C. helveticus*

* Overall mortality rate relative to the ongoing productive cycle.

## Data Availability

Not applicable.

## Supplementary Materials

The following supporting information can be downloaded at: www.mdpi.com/article/10.3390/tropicalmed7020031/s1, Table S1: List of Accession numbers used for the phylogenetic analysis.
